# Use of professional home care in persons with spinal cord injury in Switzerland: a cross-sectional study

**DOI:** 10.1186/s12913-023-10429-3

**Published:** 2023-12-12

**Authors:** Aylin Wagner, Mirjam Brach, Anke Scheel-Sailer, Manuela Friedli, Margret Hund-Georgiadis, Xavier Jordan, Martin Schubert, Armin Gemperli

**Affiliations:** 1 Swiss Paraplegic Research, Nottwil, Switzerland; 2 Swiss Paraplegic Centre, Nottwil, Switzerland; 3 Faculty of Health Sciences and Medicine, University of Lucerne, Lucerne, Switzerland; 4 ParaHelp AG, Nottwil, Switzerland; 5 REHAB Basel, Klinik für Neurorehabilitation und Paraplegiologie, Basel, Switzerland; 6 Clinique Romande de Réadaptation, Sion, Switzerland; 7 Balgrist University Hospital, Zurich, Switzerland; 8 Center of Primary and Community Care, University of Lucerne, Lucerne, Switzerland

**Keywords:** Home care, Spinal cord injury, Informal care, Cross-sectional survey

## Abstract

**Background:**

Persons with spinal cord injury (SCI) living in the community often require care. The boundaries between professional home care and informal care are blurred, and it is unclear who the typical user of home care is. The objective of this study was to describe the characteristics of persons with SCI using professional home care in Switzerland, determine the frequency of home care visits, and investigate the association of sociodemographic factors, SCI-specific characteristics, secondary health conditions, and functional independence with the use of home care.

**Methods:**

We used cross-sectional data from the 2017 community survey of the Swiss Spinal Cord Injury Cohort Study (SwiSCI). Out of 3,959 eligible individuals 1294 completed the questionnaire and were included in the analysis (response rate 33%). Using descriptive statistics, differences between home care users and non-users as well as the frequency of home care visits were investigated. The association between sociodemographic factors, SCI-specific characteristics, secondary health conditions, functional independence and the use of home care was analyzed using multivariable logistic regression. Multiple imputation was used to account for missing data.

**Results:**

Of 1,294 participants, 280 (22%) used professional home care. The median weekly professional home care duration was 6 h (Q1 = 2, Q3 = 12). More home care was used in persons with lower functional independence (Odds ratio (OR) 0.30 per 10 unit decrease in the Spinal Cord Independence Measure, 95%-Confidence interval (CI) 0.24–0.37), fewer secondary health conditions (OR 0.96 per unit Spinal Cord Injury Secondary Conditions Scale, 95%-CI 0.94–0.99), tetraplegia (OR 2.77, 95%-CI 1.92–4.00), women (OR 2.42, 95%-CI 1.70–3.43), higher age (OR 1.22 per 10 years increase, 95%-CI 1.06–1.39), living alone (OR 2.48, 95%-CI 1.53–4.03), and those receiving support from an informal caregiver (OR 1.88, 95%-CI 1.27–2.77).

**Conclusions:**

This is the first study to examine the use of professional home care from the perspective of persons with SCI in Switzerland. Lower functional independence strongly predicts increased home care use. The findings showed that professional home care complements informal care and is more likely to be used by individuals with SCI who live alone, have tetraplegia, and are female.

**Supplementary Information:**

The online version contains supplementary material available at 10.1186/s12913-023-10429-3.

## Background

Home care is the fastest-growing component of the health care system [1–3]. Factors driving this growth include the preferences of persons to age at home rather than in a hospital or nursing home, and the shift from inpatient to more cost-effective outpatient care [3]. In Switzerland, professional home care is intended for people of all age groups who require care or assistance at home. The range of services include care (e.g., care needs assessment, basic care, physical examination), assistance with activities of daily living, and household help (e.g., preparing meals, doing laundry) [4, 5]. The goals of professional home care are to help individuals to improve or sustain functioning, live with greater independence, and to assist individuals to remain at home for as long as possible, thus avoiding or delaying hospitalization or admission to long-term care institutions [6–8]. While care services prescribed by physicians are covered by social health insurance, the cost for household help must be paid by the clients themselves, potentially via private insurance covering these services [5].

Spinal cord injury (SCI) is a complex medical condition resulting from a damage to the spinal cord by accident or due to diseases (e.g., cancer, infections, vascular diseases, multiple sclerosis), necessitating often a high level of professional home care and informal care after returning home from initial rehabilitation [9]. Such care encompasses assistance with daily activities such as bathing, dressing, and household tasks as well as medical care such as wound care, or medication management. Persons with SCI experience a number of secondary health conditions such as pain, bowel and bladder regulation problems, pressure injury, muscle spasms, and respiratory complications or infections, which may require additional care and support [10]. As care for persons with SCI living in the community is complex and demanding, it often requires the coordination of care among multiple healthcare professionals and informal caregivers [11].

The boundaries between professional home care and informal care are blurred, and it is unclear who the typical user of professional home care is. The few international studies available examine the effect of specific home care programs on hospitalization and emergency department use [12–16], or costs [11, 17], offering little insight into the use of professional home care in persons with SCI. In Switzerland, Huang et al. [18, 19] investigated professional home care in persons with SCI exclusively from the perspective of family caregivers. The perspective of persons with SCI on the use of home care, as well as the determinants of home care use, have not yet been studied.

In this study, we aimed to 1) describe the characteristics of persons with SCI using professional home care in Switzerland, 2) determine the frequency of professional home care visits, and 3) investigate the association of sociodemographic factors, SCI-specific characteristics, secondary health conditions, and functional independence with the use of professional home care. We hypothesize that persons with SCI who are older, female, have no financial hardship, no support from an informal caregiver, no partner, living alone, have more severe lesion, higher levels of functional dependence, and more secondary health conditions have a higher likelihood of using professional home care.

## Methods

### Study design and study population

Cross-sectional data were obtained from the national community survey of the Swiss Spinal Cord Injury Cohort Study (SwiSCI) conducted between March 2017 and March 2018. The SwiSCI community survey was open to all Swiss residents aged over 16 years living with a traumatic or non-traumatic SCI. Individuals with congenital conditions leading to SCI, new SCI in the context of palliative care, neurodegenerative disorders, and Guillain-Barré syndrome were excluded [20]. Due to the lack of a central register for persons living with SCI in Switzerland, the study population was established based on the registries of the four Swiss specialized rehabilitation centers (Swiss Paraplegic Centre, REHAB Basel, Clinique Romande de Réadaptation, Balgrist University Hospital) and two SCI support organizations (ParaHelp, Swiss Paraplegic Association).

### Measures

#### Outcome variables

The use of professional home care was measured by asking the study participants if they currently receive support at home in daily activities, such as household chores or personal care, by a home care agency (yes/no). Information on the amount of professional home care received was collected by asking the participants about the number of home care hours received per week.

#### Independent variables

The morbidity profile included secondary health conditions and other chronic conditions (i.e., coronary heart disease, cancer, depression, sleep problems) and was assessed with the Spinal Cord Injury Secondary Conditions Scale (SCI-SCS) [21]. The study participants were asked to report the burden of 15 different health conditions over the previous 3 months on a scale of 0 (not occurred or unimportant problem), 1 (rare problem), 2 (moderate problem), or 3 (major or chronic problem). The SCI-SCS total score has a range of 0 to 45, and is based on the sum of the problem ratings. Higher scores indicate a greater number of problems with health conditions.

Functioning was evaluated using the self-report version of the Spinal Cord Independence Measure (SCIM-SR) [22]. The SCIM-SR assesses the level of independence in performing daily activities related to mobility, self-care, respiration and sphincter management. The total score ranges from 0 to 100, higher scores reflect higher functional independence. The association of the SCIM-SR subscales with the use of home care was analyzed to determine which aspect of an individual's independence is associated with greater use of care. The four subscales assess the areas of 'self-care', 'respiration & sphincter management', 'mobility in room and toilet', and 'mobility indoors and outdoors' and range from 0 to 20, 40, 10, and 30, respectively.

SCI-specific characteristics included years since injury, type of SCI (tetraplegia/paraplegia), and lesion severity (complete or incomplete loss of sensory or motor functions below the level of injury). The cause of SCI was categorized as traumatic (insult caused by an external force) or non-traumatic (injury caused by an underlying pathology).

Sociodemographic covariates included age at the time of the questionnaire, gender (men/women), place of birth (Switzerland/abroad), living situation (living alone/not living alone or in an institution), being in partnership (yes/no), and in paid employment (yes/no), and level of education (compulsory education/upper secondary level/tertiary level). Financial hardship was rated based on an assessment of its negative impact on life as either none, little, or major. Support from an informal caregiver in daily activities (yes/no) was assessed based on participants’ identification of individuals or institutions from whom they received care. The caregiver was classified as either formal (e.g., professional home care, nursing home) or informal (e.g., family, friends), depending on whether a formal agreement between caregiver and care recipient was assumed.

### Statistical analysis

All analyses were conducted using Stata version 17.0 (StataCorp, College Station, TX, USA). Descriptive statistics (i.e., frequencies, percentage, mean, standard deviation) were applied to evaluate the characteristics of the sample and to compare socioeconomic factors, SCI-specific characteristics, functional independence, and secondary health conditions of home care users with non-users. Differences between the groups were tested by χ2 test for categorical variables and t-test for continuous variables. For home care users, we also calculated the absolute and median hours of home care received per week. The Mann–Whitney U-test was used to compare the amount of home care hours between groups. Variables with a *p*-value ≤ 0.05 were reported as being statistically significant.

Logistic regression was used to assess the relationship of socioeconomic factors, SCI-specific characteristics, functional independence, secondary health conditions with home care use. Variables with a *p*-value ≤ 0.25 in univariate analyses were included in the multivariable model [23]. We report odds ratios (OR) with corresponding 95% confidence intervals (CI). Missing values were imputed with Multiple Imputation (MI) by chained equations on 20 imputed datasets, assuming that data were missing at random.

## Results

### Participants characteristics

A total of 1,294 persons with SCI completed the questionnaire (response rate 33%), including 374 (29%) women and 920 men (71%) (Table 1). The mean age of the respondents was 56 years (SD 14). About two-thirds (59%) of participants had a partner, 28% lived alone, and 45% were in paid employment. In terms of SCI-specific characteristics, 29% of the participants had tetraplegia, 32% a complete lesion, and the mean time since injury was 19 years (SD 13). Traumatic SCI was the dominant cause of SCI in 78% of cases. Overall, 56% of the participants reported being supported by an informal caregiver in daily activities.

**Table 1:** Participants characteristics, by use of home care

Characteristics	Total n (%)	Use of home care		*p*-value
		**Yes n (%)**	**No n (%)**	
Total	1294 (100.0)	280 (21.6)	1014 (78.4)	
Age, in years, mean (SD) [0]	56.4 (14.4)	59.4 (15.2)	55.6 (14.1)	***
Gender [0]				***
Men	920 (71.1)	171 (18.6)	749 (81.4)	
Women	374 (28.9)	109 (29.1)	265 (70.9)	
In partnership [0.3]				**
Yes	764 (59.0)	140 (18.3)	624 (81.7)	
No	526 (40.7)	139 (26.4)	387 (73.6)	
Living situation [1.1]				**
Living alone	366 (28.3)	103 (28.1)	263 (71.9)	
Not living alone/in an institution	914 (70.6)	174 (19.0)	740 (81.0)	
Support from an informal caregiver [0]				***
Yes	722 (55.8)	199 (27.6)	523 (72.4)	
No	572 (44.2)	81 (14.2)	491 (85.8)	
In paid employment [5.6]				***
Yes	586 (45.3)	81 (13.8)	505 (86.2)	
No	635 (49.1)	182 (28.7)	453 (71.3)	
Highest level of education [2.9]				^†^
Compulsory education	119 (9.2)	24 (20.2)	95 (79.8)	
Upper secondary level	658 (50.9)	158 (24.0)	500 (76.0)	
Tertiary level	479 (37.0)	87 (18.2)	392 (81.8)	
Financial hardship [3.0]				*
Major	92 (7.1)	21 (22.8)	71 (77.2)	
Little	201 (15.5)	59 (29.4)	142 (70.6)	
None	962 (74.3)	192 (20.0)	770 (80.0)	
Born in Switzerland [1.4]				
Yes	1,064 (82.2)	232 (21.8)	832 (78.2)	
No	212 (16.4)	47 (22.2)	165 (77.8)	
Language region [0]				
German	921 (71.2)	194 (21.1)	727 (78.9)	
French	312 (24.1)	74 (23.7)	238 (76.3)	
Italian	61 (4.7)	12 (19.7)	49 (80.3)	
Type of SCI [2.0]				***
Paraplegia	891 (68.9)	134 (15.0)	757 (85.0)	
Tetraplegia	377 (29.1)	138 (36.6)	239 (63.4)	
Lesion severity [7.9]				***
Complete	419 (32.4)	115 (27.4)	304 (72.6)	
Incomplete	773 (59.7)	137 (17.7)	636 (82.3)	
Cause of SCI [0.2]				
Traumatic	1012 (78.2)	215 (21.2)	797 (78.8)	
Nontraumatic	280 (21.6)	64 (22.9)	216 (77.1)	
Years since SCI, mean (SD) [6.1]	18.8 (13.1)	18.8 (14.6)	18.8 (12.7)	

In brackets “[]” are the percent missing values

Data are n (%) unless otherwise stated. We used Student’s t-tests for continuous variables and Chi-square-tests for categorical variables to assess univariate group differences

*n* number of observations, *SCI* Spinal Cord Injury, *SD* Standard deviation

^***^ *p* < 0.001, ** *p* < 0.01, * *p* < 0.05, † *p* < 0.1

Professional home care was used by 22% of the study participants. Study participants who were older, female (29%), without a partner (26%), living alone (28%), not in paid employment (29%), experiencing little (29%) or major financial hardship (23%), with tetraplegia (37%), complete lesion (27%), and supported by an informal caregiver (28%) reported the use of professional home care statistically significant more often than their counterparts who were younger, had a partner (18%), lived with someone (19%), were employed (14%), experienced no financial hardship (20%), had paraplegia (15%), an incomplete lesion (18%), and were not supported by an informal caregiver (14%).

**Fig. 1:**
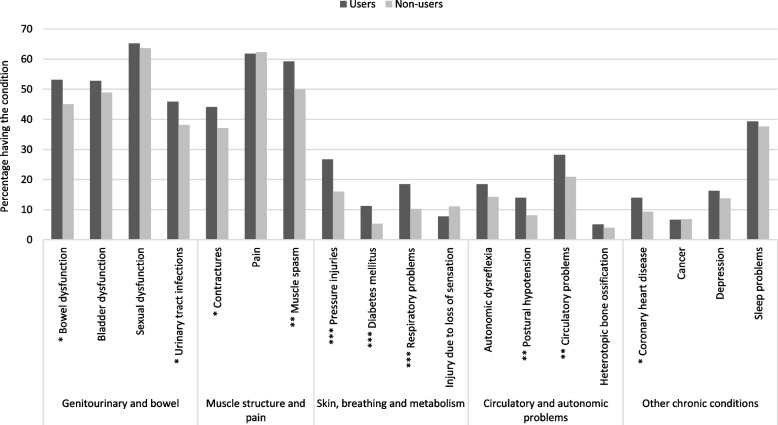
Secondary health conditions and other chronic conditions of the study participants, by use of home care (*N* = 1,294). Secondary health conditions were defined as absent when declared to be of no or rare problem, and present when declared to be of moderate or major problem during the last 3 months. *P*-values were computed using Chi-square-tests. *** *p* < 0.001, ** *p* < 0.01, * *p* < 0.05

Users and non-users of home care differed significantly regarding functional independence (Table 2). On the SCIM-SR scale from 0 to 100, persons with SCI who use home care had a score of 51 and are thus more dependent in performing daily activities than people who did not use such services, with a score of 76. Home care users had statistically significant lower scores in the areas of self-care, respiration and sphincter management, as well as mobility. Also, home care users were statistically significantly more likely to report moderate or severe problems in ten of the 19 health conditions (Fig. 1).

**Table 2:** Functional independence, by use of home care

SCIM-SR	Total Mean (SD) (*N* = 1294)	Use of home care		*p*-value
		**Yes** **Mean (SD)** **(*N* = 280)**	**No** **Mean (SD)** **(*N* = 1014)**	
Total score	70.4 (21.5)	50.8 (20.8)	75.8 (18.4)	***
Subscale self-care	16.2 (5.2)	11.3 (6.1)	17.6 (3.9)	***
Subscale respiration & sphincter management	30.4 (8.3)	23.2 (8.6)	32.4 (7.1)	***
Subscale mobility in room & toilet	7.4 (3.1)	5.7 (3.4)	7.8 (2.8)	***
Subscale mobility indoors & outdoors	15.2 (10.3)	8.4 (7.0)	17.1 (10.4)	***

*SD* Standard deviation, *SCIM-SR* Self-report version of spinal cord independence measure

*P*-values were computed using Student’s t-tests. *** *p* < 0.001

Higher scores reflect higher levels of functional independence

Total score ranges from 0–100

Subscale self-care ranges from 0–20

Subscale respiration & sphincter management ranges from 0–40

Subscale mobility in room & toilet ranges from 0–10

Subscale mobility indoors & outdoors ranges from 0–30

### Amount of home care

**Fig. 2:**
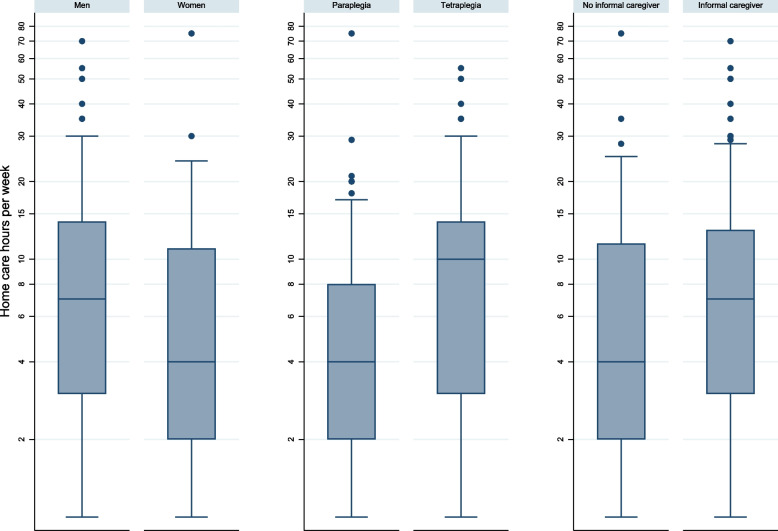
Amount of home care hours received per week in persons with spinal cord injury (*N* = 252). Mann–Whitney U-test was used for statistical comparison of the groups. Gender *p* < 0.001, SCI type *p* < 0.001, support from informal caregiver *p* = 0.049

Of the 280 persons using professional home care, 252 (90%) reported hours (h) of home care received per week. The median hours per week were 6 h (Q_1_ = 2, Q_3_ = 12), but less in women, persons with paraplegia and persons without an informal caregiver (all median 4 h/week) (Fig. 2).

### Factors associated with home care use

Table 3 shows the factors associated with the use of professional home care in persons with SCI (crude OR Supplementary Table 1). Excluded from the multivariable model, due to *p* > 0.25 in univariate analyses, were place of birth, language region, educational level, financial hardship, cause of SCI, and years since injury. Persons with higher functional independence (OR 0.30 per 10 unit increase in SCIM-SR, 95%-CI 0.24–0.37) and more secondary health conditions (OR 0.96 per unit SCI-SCS, 95%-CI 0.94–0.99) were less likely to use home care. Home care was used more likely in women (OR 2.42, 95%-CI 1.70–3.43), persons of higher age (OR 1.22 per 10 years increase, 95%-CI 1.06–1.39), with tetraplegia (OR 2.77, 95%-CI 1.92–4.00), living alone (OR 2.48, 95%-CI 1.53–4.03), and receiving support from an informal caregiver (OR 1.88, 95%-CI 1.27–2.77).

**Table 3:** Use of home care – adjusted logistic regression model

Predictor	OR	95%-CI
SCIM-SR (per 10 units)	0.30***	0.24–0.37
SCI-SCS	0.96**	0.94–0.99
Type of SCI (ref = Paraplegia)		
Tetraplegia	2.77***	1.92–4.00
Lesion severity (ref = Complete)		
Incomplete	0.95	0.65–1.39
Gender (ref = Men)		
Women	2.42***	1.70–3.43
Age (per 10 years)	1.22**	1.06–1.39
In partnership (ref = No)		
Yes	0.76	0.47–1.24
Living situation (ref = Not living alone)		
Living alone	2.48***	1.53–4.03
In paid employment (ref = No)		
Yes	0.77	0.51–1.16
Support from an informal caregiver (ref = No)		
Yes	1.88**	1.27–2.77

*ref* Reference category, *OR* Odds ratio, *95%-CI* 95% confidence interval

^***^ *p* < 0.001, ** *p* < 0.01

SCIM-SR: scale from 0–100, higher numbers indicate higher functional independence

SCI-SCS: Spinal Cord Injury Secondary Conditions Scale from 0 to 45. Higher scores reflect a greater number of problems with secondary health conditions

## Discussion

In our sample of persons with SCI living in the community in Switzerland, about one of five uses professional home care. The median weekly amount of home care received was 6 h, with statistically significant differences between genders, SCI type and whether informal caregiver support was received. Several sociodemographic characteristics, SCI type, secondary health conditions and functional independence were associated with the use of home care.

As hypothesized, functional independence is a strong predictor of home care use: per 10-unit decrease in the SCIM-SR (higher scores indicate higher functional independence), the odds of using home care increased by 233%. It is not surprising that persons with SCI who have lower functional independence need more support in daily activities, and our observations corroborate those from other research, for example, by Weitzenkamp et al. [24] who examined the predictors of personal care assistance use in persons with SCI in the United States (US), or by Huang et al. [18] who surveyed informal caregivers of persons with SCI in Switzerland. Also, in the general population, several studies identified difficulty in one or more activities of daily living as one of the strongest predictors of home care use [25–31].

The hypothesis that persons are more likely to use home care because of secondary health conditions had to be rejected. Although persons with SCI who used home care significantly more often reported moderate or major problems regarding some specific secondary health conditions, we found a negative and significant effect of the SCI-SCS scale in the multivariable model, meaning those reporting more secondary health conditions were less likely to use home care. This finding was not expected and it is likely that secondary health conditions alone are not the main driver for the use of home care. Rather, it is the complex interrelationships between functioning, age-related factors, and the development and trajectory of secondary health conditions [32]. The possibility that individuals with many secondary health conditions are more likely to seek a care solution outside the official home care program can be ruled out, given the structure of the Swiss health care system. It is important to note that these results are specific to person-reported secondary health conditions, whereas an analysis based on objective measures of secondary health conditions might have found a different effect. Thus, our analysis specifically addressed the person's perspective, which may differ from that of health care providers. Future research looking into these interrelationships is needed to fully understand the relationship between secondary health conditions and the use of professional home care.

Persons with SCI who received support from an informal caregiver had 88% higher odds using home care, and they also received more hours of home care per week than those without informal caregivers. This suggests that professional home care does not replace informal care but rather complements it, particularly in more complex care situations. In addition, there is evidence for what is known as 'bridging processes' [33], in which informal care facilitates professional care. Meaning that informal caregivers can help identify the need for professional home care, navigate the care system, and connect individuals with SCI with professional home care providers, resulting in the (timely) initiation of professional home care. Our findings are consistent with Huang et al. [19] who found that the total time investment of family caregivers of persons with SCI in Switzerland remained largely unchanged, despite the increase in professional home care. Studies conducted in older European populations [25, 34, 35] have found that higher professional home care provision leads to an increase in informal care utilization. However, there is mixed evidence, as other studies [36, 37] have found the exact opposite – that informal care and professional home care are substitutes, i.e., the utilization of professional home care decreases the amount of informal care. However, the results also showed that individuals with SCI who live alone had 148% higher odds of using home care services, indicating that professional home care services can substitute for informal care in certain circumstances, such as in absence of a 'potential' informal caregiver residing in the same household.

Regarding socioeconomic characteristics, education level, financial hardship, language region, and place of birth were not part of the final model since they were not statistically significant predictors of home care use at the univariate stage. However, we observed a significant association with both gender and age. Specifically, the odds of using home care increased by 22% for every 10-year increase in age. This is expected as older individuals may have more difficulties performing daily tasks and may require more assistance to live at home. Additionally, person with SCI experience accelerated ageing and therefore accelerated dependency [38].

Women with SCI had 142% higher odds of using home care compared to men, which is consistent with previous research in Denmark [39] and the US [40]. In the general population, studies also showed that women are more likely to pay for professional home care services, whereas men rely more heavily on their spouses for care [9, 41–43]. However, despite their greater need, we found that women received fewer hours of home care per week than men. This is inconsistent with Huang et al. [19] who observed that larger proportion of professional home care was requested for women with SCI living in Switzerland.

### Strengths and limitations

To our knowledge, this study is the first to assess determinants of home care use in Switzerland from the perspective of persons with SCI. A notable strength of this study is its large sample size and comprehensiveness of investigation, which was conducted on a national level and can be considered representative of the Swiss SCI population living in the community [20].

Nevertheless, this study has some limitations. First, the cross-sectional design of the study precluded an examination of home care use over time and causal inferences could not be made. Second, we were unable to differentiate between the types of home care services, such as care services or household help, which may have different determinants of utilization. Thirdly, we could not control for insurance coverage, as the data did not reliably determine who covered the cost for home care (e.g., private insurance, disability insurance). It is worth noting, however, that insurance coverage is only a barrier to the use of household help, as these services – unlike the care services – are not covered by social health insurance [5]. Finally, the study is specific about individuals with SCI living in the community in Switzerland, and generalizability to other countries is limited. Switzerland has one of the highest health care expenditures of any country and offers its residents universal access to the world's largest health care workforce. Thus, the results are an illustration of service utilization in the context of widely guaranteed access to home care.

## Conclusions

Functional independence was found to be a strong predictor of home care use, with those with lower functional independence requiring more support. The study indicated that professional home care complements informal care and is more likely to be used by individuals with SCI who live alone, have tetraplegia, and are female. Women are more likely to use home care services, but for fewer hours than men. The reasons for this difference, including the influence of medical and social factors, family dynamics, and expectations, remain unclear. Future research is needed to fully understand the complex interplay between informal care and professional home care and the gender differences in order to tailor home care services to individual needs and circumstances.

### Supplementary Information

**Additional file 1. **

## Data Availability

Owing to our commitment to SwiSCI study participants and their privacy, datasets generated during the current study are not made publicly available but can be provided by the SwiSCI Study Center based on reasonable request (contact@swisci.ch).

## Abbreviations

CI: Confidence interval
OR: Odds ratio
SCI: Spinal cord injury
SCI-SCS: Spinal Cord Injury Secondary Conditions Scale
SCIM-SR: Spinal Cord Independence Measure-Self Report
SwiSCI: Swiss Spinal Cord Injury Cohort Study
US: United States
