# Supplementary material for: Use of professional home care in persons with spinal cord injury in Switzerland: a cross-sectional study
Source: BMC Health Serv Res. 2023 Dec 12;23:1393. doi: 10.1186/s12913-023-10429-3 (PMC10714621; doi:10.1186/s12913-023-10429-3)
Supplement: Supplementary file 1 — Additional file 1. [file 12913_2023_10429_MOESM1_ESM.docx]

Supplementary table 1. Use of home care – Unadjusted logistic regression model

| **Predictor** | **OR** | **95%-CI** |
| --- | --- | --- |
| SCIM-SR (per 10 units) | 0.26*** | 0.21-0.32 |
| SCI-SCS | 1.04*** | 1.02-1.07 |
| Type of SCI (ref = Paraplegia) |  |  |
| Tetraplegia | 3.26*** | 2.47-4.31 |
| Lesion severity (ref = Complete) |  |  |
| Incomplete | 0.57*** | 0.43-0.76 |
| Gender (ref = Men) |  |  |
| Women | 1.80*** | 1.36-2.38 |
| Age (per 10 years) | 1.20*** | 1.10-1.32 |
| In partnership (ref = No) |  |  |
| Yes | 0.62** | 0.48- 0.82 |
| Living situation (ref = Not living alone) |  |  |
| Living alone | 1.67*** | 1.26- 2.21 |
| In paid employment (ref = No) |  |  |
| Yes | 0.40*** | 0.30-0.53 |
| Support from an informal caregiver (ref = No) |  |  |
| Yes | 2.31*** | 1.73-3.07 |

ref = reference category; OR = odds ratio; 95%-CI = 95% confidence interval.

*** p < 0.001, ** p < 0.01.

SCIM-SR: scale from 0-100, higher numbers indicate higher functional independence.

SCI-SCS: Spinal Cord Injury Secondary Conditions Scale from 0 to 45. Higher scores reflect a greater number of problems with secondary health conditions.
